# Age-associated changes in the transcriptomes of non-cultured adipose-derived stem cells from young and old mice assessed via single-cell transcriptome analysis

**DOI:** 10.1371/journal.pone.0242171

**Published:** 2020-11-25

**Authors:** Yuta Doshida, Haruka Sano, Sadahiro Iwabuchi, Toshiro Aigaki, Masayuki Yoshida, Shinichi Hashimoto, Akihito Ishigami

**Affiliations:** 1 Molecular Regulation of Aging, Tokyo Metropolitan Institute of Gerontology, Tokyo, Japan; 2 Department of Biological Sciences, Tokyo Metropolitan University, Tokyo, Japan; 3 Department of Life Science and Bioethics, Graduate School of Medicine, Tokyo Medical and Dental University, Tokyo, Japan; 4 Department of Molecular Pathophysiology, Institute of Advanced Medicine, Wakayama Medical University, Wakayama, Japan; Universita degli Studi Di Cagliari, ITALY

## Abstract

Adipose-derived stem cells (ASCs) exhibit self-renewal and pluripotency. The differentiation potency of ASCs has been reported to deteriorate with aging; however, relevant studies used ASCs that were isolated and subcultured several times. It is still unclear whether subcultured ASCs accurately reflect the *in vivo* state. To address this question, we used freshly isolated stromal vascular fractions (SVFs) and performed comprehensive single-cell transcriptome analysis. In this study, we identified three cell populations as putative ASC candidates in SVFs and three novel ASC-related genes: *Adamts7*, *Snai2*, and *Tgfbr1*, that are reported to be negative regulators of cell differentiation. Moreover, we identified age-associated high gene expression levels of *Adamts7*, *Egfr*, and *Igfbp4* in the earliest differentiation stage of ASCs. These results suggest that aging may make it impossible to maintain the stringency of the regulation of the expression of some genes related to ASC differentiation.

## Introduction

Adipose-derived stem cells (ASCs) are highly convenient multipotent mesenchymal stem cells for clinical application in human regenerative medicine because they are easily isolated from adipose tissues [1–4]. Isolated ASCs can be stably subcultured in large quantities at the same time and be induced to differentiate into adipogenic, osteogenic, chondrogenic, and angiogenic cells [3].

Many patients who receive regenerative medicine are elderly people, so the isolation of ASCs from adipose tissue is likely to be more common in elderly people. Therefore, it is very important to ensure that ASCs from elderly people are comparable to those from young people regarding cell characteristics such as differentiation ability and the maintenance of stemness and with respect to more specific gene expression patterns in ASCs.

There have been several reports comparing ASCs from elderly people with ASCs from young people [5–8]. Liu *et al*. [5] compared ASCs from three different age groups (children, young adults, and elderly people) and revealed that ASCs from elderly people exhibited a low differentiation potential and poor migration abilities compared with ASCs from young people. Moreover, Maredziak *et al*. [7] reported that ASCs from elderly people presented decreased proliferation rates, decreased chondrogenic and osteogenic potential, and increased senescence features. Thus, ASCs from elderly people show reduced differentiation and proliferation abilities compared with ASCs from young people. However, these studies used ASCs that had been isolated from adipose tissues and subcultured several times. To minimize the effect of subculture of ASCs on gene expression, we used isolated stromal vascular fractions (SVFs) including ASCs that were not cultured or passaged and performed comprehensive single-cell transcriptome analysis to identify ASCs in SVFs.

In this study, we identified ASCs in SVFs via single-cell transcriptome analysis and examined the age-associated changes in the transcriptome of ASCs from young and old mice. We identified age-associated changes in gene expression in non-cultured ASCs for the first time. These findings will greatly contribute to the development of regenerative medicine.

## Materials and methods

### Animals

Animal experiments were conducted in accordance with the animal care and use protocol approved by the Institutional Animal Care and Use Committee of the Tokyo Metropolitan Institute of Gerontology (TMIG, Tokyo, Japan) (Permit Number: 18028) and the Guidelines for the Care and Use of Laboratory Animals of TMIG. Male mice of the C57BL/6NCr strain at 6 and 29 months of age were obtained from the animal facility of TMIG. All mice were fed CRF-1 (Oriental Yeast Ltd., Tokyo, Japan) [9] *ad libitum*. Throughout the experiments, the animals were maintained under a 12-h light/dark cycle in a controlled environment. The number of animals that was used was kept to the minimum necessary for a meaningful interpretation of the data, and animal discomfort was kept to a minimum.

### Isolation of the SVF

SVF was isolated enzymatically from excised fat tissue by digestion with collagenase using the method of Sugii *et al*. [4], as illustrated in S1 Fig. Briefly, epididymal adipose tissues were removed from one 6-month-old and one 29-month-old male mouse that had been perfused with phosphate-buffered saline through the left ventricle to remove hematopoietic cells from epididymal adipose tissues and digested by incubation with 2 mg/mL type I collagenase (Gibco, Thermo Fisher Scientific, Waltham, MA, USA) in Hank’s balanced salt solution containing 1% bovine serum albumin (Sigma-Aldrich, St. Louis, MO, USA), 200 nM adenosine (Sigma-Aldrich), and 50 μg/mL glucose (VWR International, Radnor, PA, USA) at 37°C for 1 h with gentle agitation. After centrifugation for 5 min at 1,450 rpm, floating mature adipocytes were removed. The cell pellet was suspended in Hank’s balanced salt solution, followed by filtration through a 100 μm nylon mesh filter (Corning, Corning, NY, USA) and then centrifuged at 1,450 rpm for 5 min. Thereafter, the pelleted cells were treated with ammonium-chloride-potassium lysing buffer (Lonza, Alpharetta, GA, USA) to remove remaining erythrocytes, followed by centrifugation at 1,450 rpm for 5 min. The cell pellet was washed with phosphate-buffered saline and then used as SVF for single-cell transcriptome analysis.

### Next-generation 1-cell sequencing (Nx1-seq)

Single-cell transcriptome analysis, which we referred to as Nx1-seq, was performed as described previously [10] and is illustrated in S2 Fig. This Nx1-seq approach can provide digital gene expression data for hundreds or thousands of single cells. Briefly, poly(dT) barcoded beads were first added to a microwell slide (1.6 × 10^5^ wells, 2 × 2 mm) at 1 bead/well. Cells were allowed to settle into the 20-pL wells of a polydimethylsiloxane slide via gravity. The slides were covered with a dialysis membrane and then incubated with a cell lysis solution containing detergents. After lysis, poly(dT) barcoded beads with bound cellular mRNA were collected in a microtube, and cDNA was synthesized with reverse transcriptase. The cDNA was stored at −30°C until use.

### Generation of the next-generation sequencing library

The cDNA amplified by PCR was fragmented by an M220 Focused-ultrasonicator (Covaris Inc., Woburn, MS, USA), and then a sequence-ready library was produced by following the instructions of the Illumina TruSeq^™^ library prep kit (Illumina, San Diego, CA, USA). The quality and quantity of the library were confirmed with an Agilent 4200 TapeStation (Agilent, Santa Clara, CA, USA) and Roche^®^ KAPA Library Quantification Kits (Merck KGaA, Darmstadt, Germany). The single-cell RNA sequencing results were read with a NextSeq 500/550 High Output v2 Kit (75 cycles) (Illumina), and we used the paired-end sequencing mode (read1 25 cycles, read2 60 cycles) with custom primers based on our bar-coded beads [10].

### Read alignment and gene expression quantification

The Nx1-seq data were aligned and annotated as described previously [10]. Briefly, barcode sequences were extracted from the read 1 fastq files. The read 2 fastq files, which included each cell mRNA, were directly aligned to Refseq transcript sequences (ftp://ftp.ncbi.nih.gov/refseq/H_sapiens/mRNA_Prot) using bowtie 2.2.6 [11]. The aligned reads were linked to their paired extracted barcode sequences. By counting mapped reads per barcode, the gene count data in individual cells were obtained and the transcripts per million of each gene were calculated in each cell.

The gene count data in individual cells obtained from SVF were analyzed using Seurat 2.4 software [12]. We implemented a series of quality controls as follows: First, any gene expressed by less than 3 cells at less than 3 counts per million were removed. Second, we filtered out the cells that satisfied the following conditions: the number of genes detected was less than 400 or more than 8,000, or the percentage of mitochondrial gene counts was more than 5. After filtering, we finally obtained 1,286 cells and 19,936 genes for further analysis. Next, we classified cells into 11 clusters using the t-distributed stochastic neighbor embedding (t-SNE) method [13].

### Gene ontology (GO) terms

The GO terms of genes were searched using the functional annotation of the Database for Annotation, Visualization, and Integrated Discovery (DAVID) version 6.8 [14, 15]. Official gene symbols were uploaded to DAVID. We also used DAVID to search GO terms in the module and extracted gene sets that were assigned GO terms whose *p*-values were lower than 0.05 in the GOTERM_BP_DIRECT category.

### Pseudotime analysis

The R package Monocle 3 version 0.2.0 was used to search for highly expressed genes using R version 3.6.1 [16]. The data were treated by preprocessing, reduction of dimensions, clustering, and plotting to uniform manifold approximation and projection (UMAP) [17]. Monocle 3 provided a path in the UMAP space using a principal graph-embedding procedure based on the SimplePPT algorithm [18, 19]. Pseudotime was calculated based on Euclidean distance in the UMAP space after artificially setting the start and end points of the pseudotime course [20]. Genes whose expression changed significantly along with pseudotime were extracted using Moran’s I test for spatial autocorrelation [21] and were clustered into modules. The modules consisted of genes whose expression patterns were similar. Heat maps were visualized for the expression of every module and hierarchical clustering analysis.

### Gene set enrichment analysis (GSEA)

GSEA version 4.0.2 was applied using two databases, GO (c5.all) and REACTOME (c2.all) [22, 23]. Gene expression data presented as gene symbols were converted to the Mouse Genome Informatics (MGI) ID [24] provided by Jackson Laboratory (Bar Harbor, ME, USA) based on the GRCm38 dataset in the Ensembl Gene 98 database using BioMart provided by Europe’s flagship laboratory for the life sciences, the European Bioinformatics Institute (EMBL-EBI). After ranking and rearranging the genes based on expression data using the Signal2Noise metric, the enrichment scores of the gene sets were calculated using a weighted scoring scheme. NES was calculated with 1,000 permutations. Gene sets with nominal p-values less than 0.05 were selected.

### Protein–protein interaction (PPI) analysis

STRING version 11.0 was used to analyze protein–protein association networks with high confidence (more than 7.0 confidence score) [25]. Four evidence data, such as (i) co-expression, (ii) text-mining, (iii) biochemical/genetic data (“experiments”), and (iv) previously curated pathway and protein-complex knowledge (“databases”) of STRING were used to calculate the interaction scores of each network edge.

### Accession numbers

The Nx1-seq data have been deposited in the DNA Databank of Japan (DDBJ) with the accession number DRA009598.

### Data availability

The datasets generated and/or analyzed during the current study are available from the corresponding author upon reasonable request.

## Results

### Single-cell transcriptome analysis of mouse SVFs

**Fig 1:**
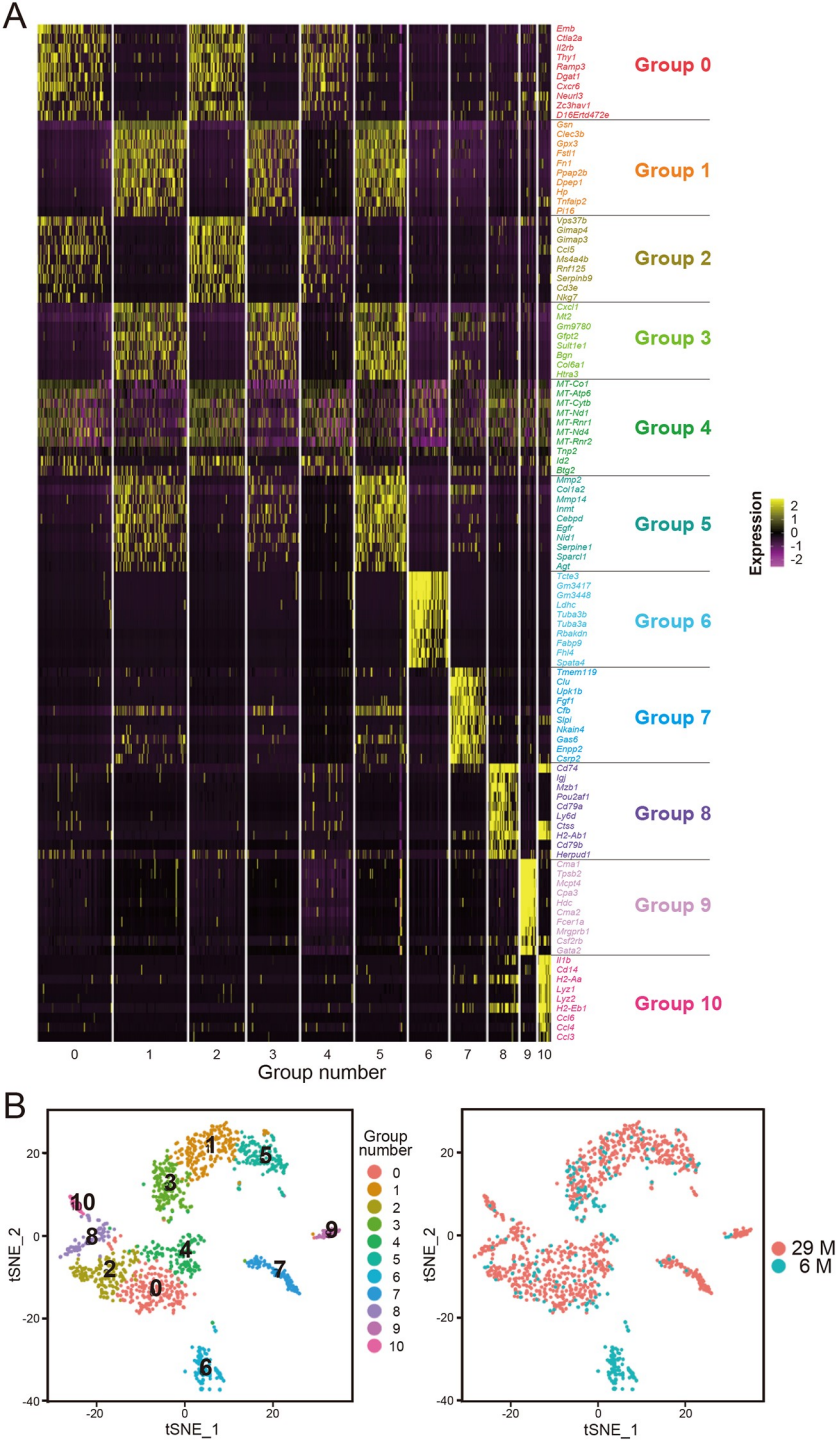
Clustering analysis and t-SNE plots of transcriptome data obtained from 6-month-old (young) and 29-month-old (old) mice. (A) SVF cell populations from young (cell number, n = 271) and old (cell number, n = 1015) mice were classified into eleven numbered groups (Groups 0 to 10) using the package Seurat version 2.4. A heat map for the genes that were differentially expressed in each of the 11 groups is shown. (B) The t-SNE plot of SVFs from young and old mice is colored according to the group number (left) and age (right).

To identify and compare the ASCs in SVFs from 6-month-old (young) and 29-month-old (old) mice, we performed comprehensive single-cell transcriptome analysis. In this study, we used one young and one old mouse to eliminate individual differences. SVFs from mouse epididymal white adipose tissues were isolated by collagenase digestion and centrifugation to remove low-density fatty mature adipocytes, as described in the Methods and illustrated in S1 Fig. The isolated SVFs were subjected to our previously developed single-cell transcriptome analysis using bar-coded beads and processed microwells, which we refer to as Nx1-seq [10], as illustrated in S2 Fig. To identify ASCs in SVFs, we first performed clustering analysis and obtained t-SNE plots using the combined single-cell gene expression data from the two age groups (Fig 1A and 1B). The cell populations of the SVFs were classified into eleven groups (Groups 0 to 10).

### Identification of ASC candidates in SVFs

**Fig 2:**
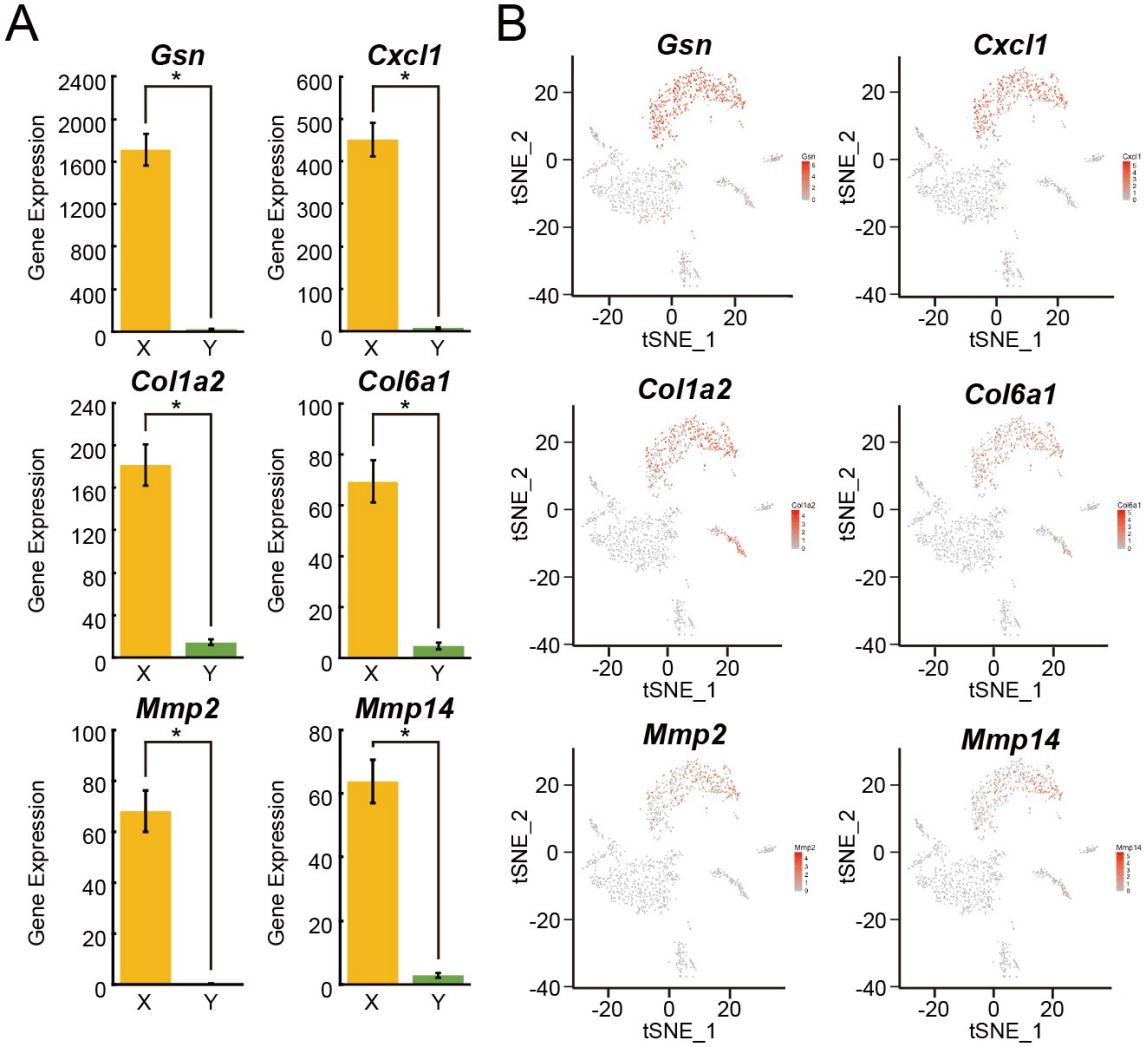
ASC marker gene expression in SVFs. (A) The gene expression levels of *Gsn*, *Cxcl1*, *Col1a2*, *Col6a1*, *Mmp2*, and *Mmp14* in Groups 1, 3, and 5 combined (as indicated in X) (cell number, n = 461) and eight other groups (as indicated in Y) (cell number, n = 825) are shown. (B) Heat maps of *Gsn*, *Cxcl1*, *Col1a2*, *Col6a1*, *Mmp2*, and *Mmp14* in t-SNE plots are shown. Values are presented as the means ± standard error of the means (SEMs). The statistical analysis was performed using the two-tailed Welch’s *t*-test. *p<0.001.

To identify the cell types present in these eleven groups, we searched GO terms using the functional annotation of the DAVID version 6.8 [14, 15] (S1 and S2 Tables). We inferred the corresponding cell types of these eleven groups as shown in S2 Table, and we considered Groups 1, 3, and 5 to be major ASC candidates because 18 genes (including gelsolin (*Gsn*), C-type lectin domain family 3, member b (*Clec3b*), glutathione peroxidase 3 (*Gpx3*), follistatin-like 1 (*Fstl1*), phosphatidic acid phosphatase type 2B (*Ppap2b*), dipeptidase 1 (*Dpep1*), haptoglobin (*Hp*), peptidase inhibitor 16 (*Pi16*), chemokine (C-X-C motif) ligand 1 (*Cxcl1*), sulfotransferase family 1E, member 1 (*Sult1e1*), biglycan (*Bgn*), HtrA serine peptidase 3 (*Htra3*), matrix metallopeptidase 2 (*Mmp2*), indolethylamine N-methyltransferase (*Inmt*), nidogen 1 (*Nid1*), SPARC-like 1 (*Sparcl1*) and, angiotensinogen (serpin peptidase inhibitor, clade A, member 8) (*Agt*)) out of the 28 genes identified in these groups were listed as genes that are highly expressed in ASCs in a report by Burl *et al*. [26] (Fig 1 and S2 Table). Moreover, typical ASC marker genes such as *Gsn*, *Cxcl1*, collagen, type I, alpha 2 (*Col1a2*), collagen, type VI, alpha 1 (*Col6a1*), *Mmp2*, and matrix metallopeptidase 14 (membrane-inserted) (*Mmp14*) [27], showed high expression levels in Groups 1, 3, and 5 combined (as indicated in X in Fig 2A) compared with the other eight groups (as indicated in Y in Fig 2A) (Fig 2A and 2B). From these results, we concluded that Groups 1, 3, and 5 were the major ASC candidates in SVFs.

### Age-associated changes in the percentage of cells positive for ASC marker gene expression

**Fig 3:**
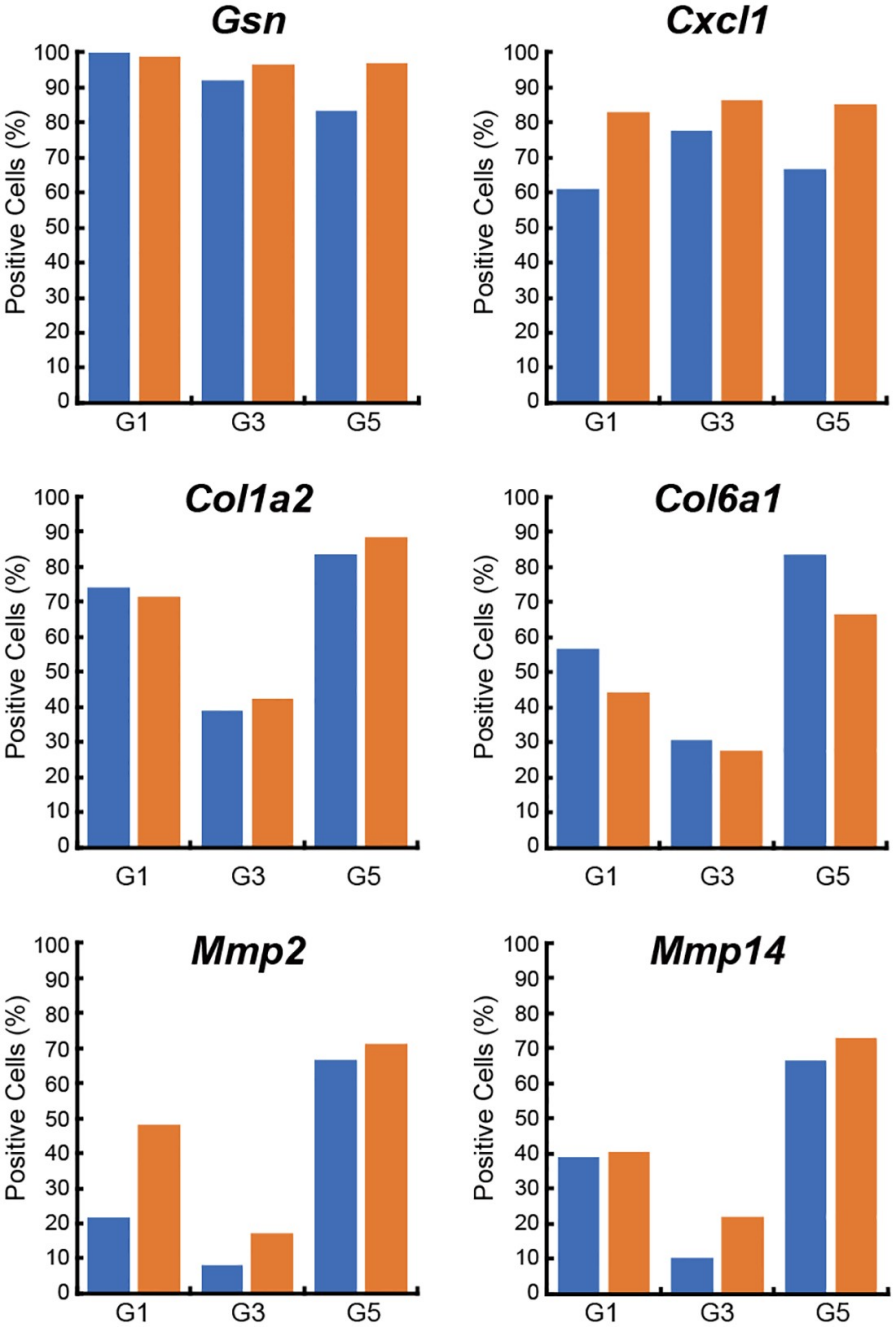
Age-associated changes in ASCs expressing ASC marker genes. The percentages of cells positive for the expression of *Gsn*, *Cxcl1*, *Col1a2*, *Col6a1*, *Mmp2*, and *Mmp14* in Groups 1, 3, and 5 from young (blue) and old (orange) mice are shown. G1: Group 1 (cell number, n = 191), G3: Group 3 (cell number, n = 136), G5: Group 5 (cell number, n = 134).

The total cells of Groups 1, 3, and 5 from young and old mice accounted for 50% and 37% of the SVF cells, respectively. We next examined the age-associated change in the percentage of cells positive for the expression of ASC marker genes, such as *Gsn*, *Cxcl1*, *Col1a2*, *Col6a1*, *Mmp2*, and *Mmp14*, in Groups 1, 3, and 5 (Fig 3). In Group 1, the percentages of *Cxcl1-* and *Mmp2*-expressing cells in old mice were higher than those in young mice. Moreover, in Group 5, the percentage of *Gsn*-expressing cells in old mice was higher than that in young mice. However, no age-associated changes were observed in the percentages of *Col1a2-*, *Col6a1-*, and *Mmp14*-expressing cells between young and old mice.

### Differentiation stages of ASCs

**Fig 4:**
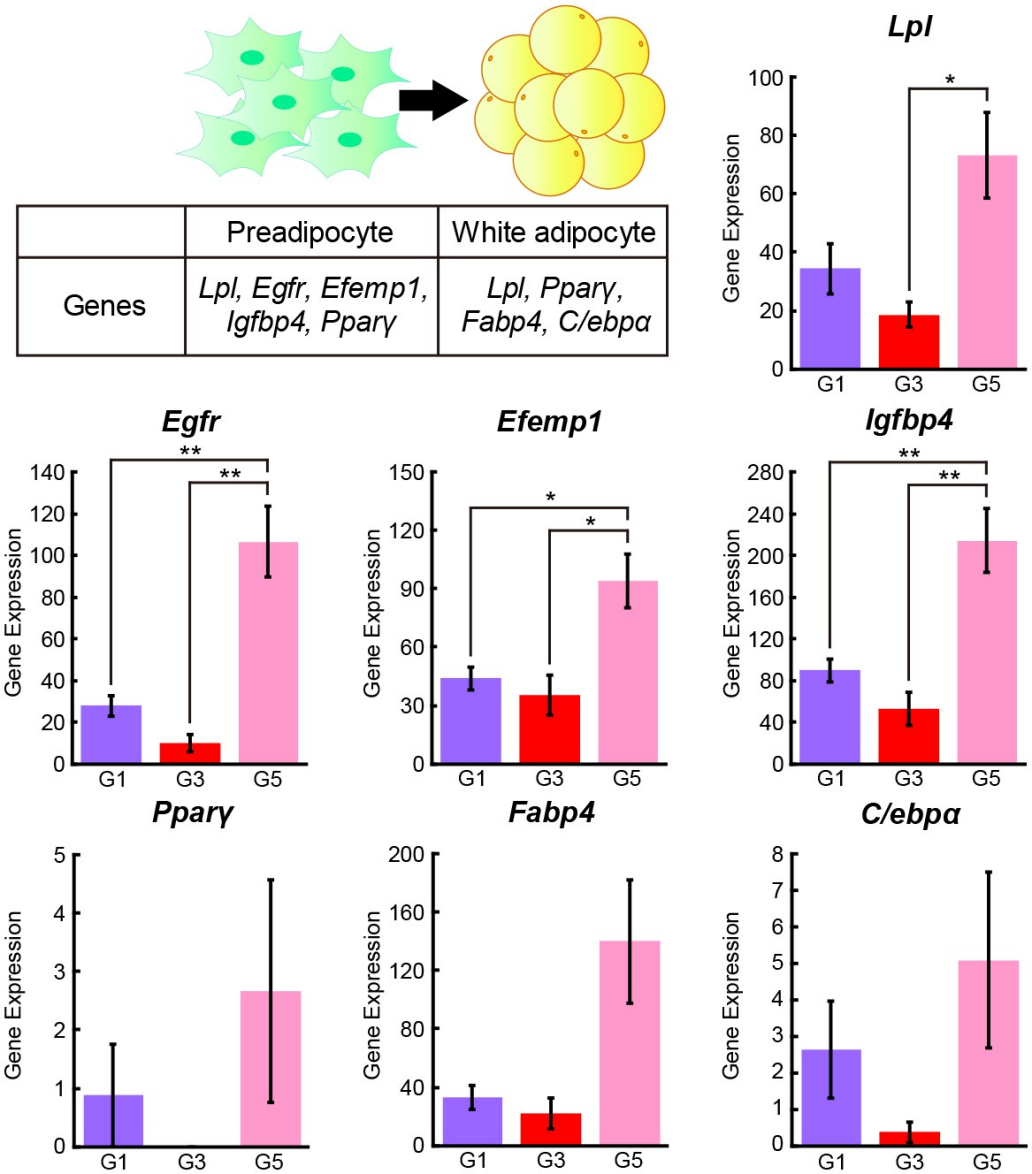
Preadipocyte and white adipocyte marker gene expression among Groups 1, 3, and 5. The gene expression levels of *Lpl*, *Egfr*, *Efemp1*, *Igfbp4*, *Pparγ*, *Fabp4*, and *C/ebpα* in Groups 1 (purple), 3 (red), and 5 (magenta) are shown. G1: Group 1 (cell number, n = 191), G3: Group 3 (cell number, n = 136), G5: Group 5 (cell number, n = 134). Values are presented as the means ± SEMs. The statistical analysis was performed using one-way analysis of variance followed by the Tukey-Kramer test. *p<0.05, **p<0.01.

To clarify and compare the differentiation stages of Groups 1, 3, and 5, we examined the expression levels of genes expressed in preadipocytes and white adipocytes, such as lipoprotein lipase (*Lpl*), epidermal growth factor receptor (*Egfr*), epidermal growth factor-containing fibulin-like extracellular matrix protein 1 (*Efemp1*), insulin-like growth factor binding protein 4 (*Igfbp4*), peroxisome proliferator activated receptor gamma (*Pparγ*), fatty acid binding protein 4, adipocyte (*Fabp4*), and CCAAT/enhancer-binding protein (C/EBP), alpha (C*/ebpα*) [28] (Fig 4). The expression levels of *Egfr*, *Efemp1*, and *Igfbp4* in Group 1 were significantly lower than those in Group 5. Moreover, the expression levels of *Lpl*, *Egfr*, *Efemp1*, and *Igfbp4* in Group 3 were significantly lower than those in Group 5. However, no significant difference was observed in any of the genes examined between Groups 1 and 3. Moreover, the expression levels of *Lpl*, *Egfr*, *Efemp1*, *Igfbp4*, *Pparγ*, *Fabp4*, and *C/ebpα* in Group 3 exhibited the greatest reduction compared with those in Groups 1 and 5, but no significant differences were observed. *Pparγ* expression was not detected in Group 3. Based on these results, we considered Group 3 to represent the earliest differentiation stage, followed by Group 1, while Group 5 represented the most advanced differentiation stage of ASCs.

### Pseudotime analysis

**Fig 5:**
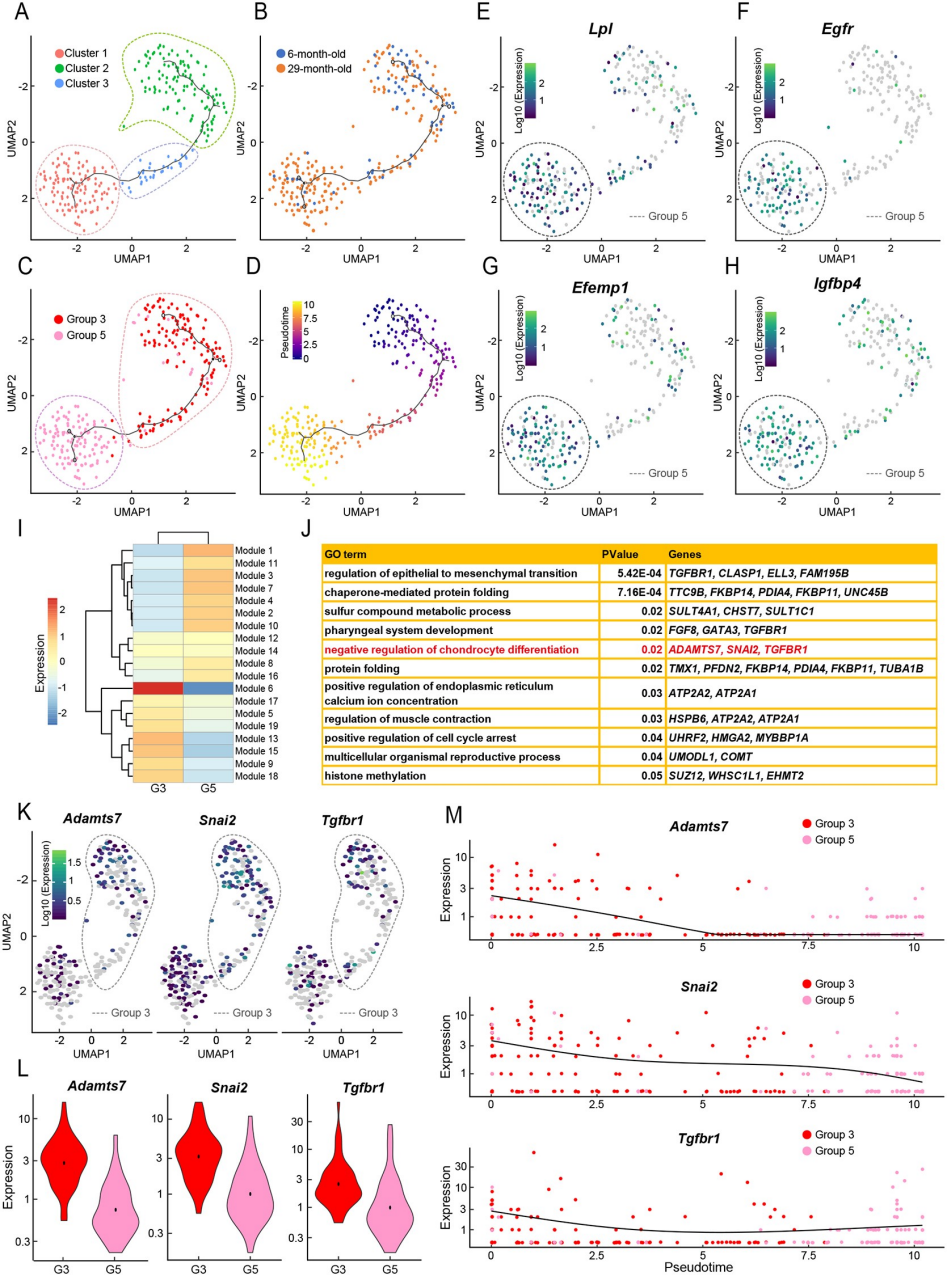
Pseudotime analysis using Monocle 3. (A-D) The data of Group 3 (cell number, n = 136) and Group 5 (cell number, n = 134) from young and old mice were reanalyzed using Monocle 3. Black lines in the plot show the paths of pseudotime. UMAP plots are colored according to three clusters (A), age (B), groups (C), and pseudotime (D). (E-H) Heat maps showing the Log10 expression levels of *Lpl*, *Egfr*, *Efemp1*, and *Igfbp4* in the UMAP plot. Gray dotted lines indicate the Group 5 area. (I) The heat maps of the hierarchical cluster analysis and expression of modules between Group 3 (G3) and Group 5 (G5) are shown. The modules consist of genes clustered according to the correlation of expression levels. Genes whose expression changed significantly over pseudotime were extracted using Moran’s I test for spatial autocorrelation (q-value < 0.05). (J) GO terms of module 6 genes searched using DAVID (p-value < 0.05). (K) Heat maps showing the Log10 expression levels of *Adamts7*, *Snai2*, and *Tgfbr1* in the UMAP plot. Gray dotted lines indicate the Group 3 area. (L) Violin plots showing the distribution of *Adamts7*, *Snai2*, and *Tgfbr1* expression in Group 3 (red) and Group 5 (magenta). (M) Kinetic plots showing the expression of *Adamts7*, *Snai2*, and *Tgfbr1* over pseudotime are shown.

To investigate the differentiation stages in more detail, we used transcriptome data from only Groups 3 (assumed to represent the earliest stage of ASCs) and 5 (assumed to represent the most advanced stage of ASCs) and reanalyzed these data using Monocle 3 [16]. After the reduction of dimensions by UMAP [17] and clustering analysis, the cell populations were classified into three numbered clusters (Clusters 1, 2, and 3) (Fig 5A). In addition, the cell populations from young and old mice were individually plotted (Fig 5B). The UMAP plots showed that Cluster 1 included most of the transcriptome data from Group 5, while Clusters 2 and 3 included most of the transcriptome data from Group 3 (Fig 5A and 5C). Furthermore, heat map plots of preadipocyte marker genes such as *Lpl*, *Egfr*, *Efemp1*, and *Igfbp4* showed higher expression levels in group 5 than in group 3 (Fig 5E–5H). Therefore, we inferred that the differentiation stages of ASCs progressed from Group 3 to Group 5, and the calculated pseudotime indicated that Group 3 represented the starting point (Fig 5D).

### Search for specific genes associated with pseudotime

We next explored specific genes whose expression levels changed along with the elapsed pseudotime using Moran’s I test for spatial autocorrelation [21], and the results are listed in S3 Table. We further performed hierarchical clustering analysis of the modules with similar gene expression patterns based on expression correlations (Fig 5I). In particular, Module 6 showed remarkably higher expression levels in Group 3 than in Group 5 (Fig 5I and S4 Table). Moreover, the functional annotation of DAVID version 6.8 [14, 15] provided GO terms from Module 6 and identified three interesting genes related to cell differentiation: a disintegrin-like and metallopeptidase (reprolysin type) with thrombospondin type 1 motif, 7 (*Adamts7*) gene, a snail family zinc finger 2 (*Snai2*) gene, and a transforming growth factor, beta receptor I (*Tgfbr1*) gene (GO term, negative regulation of chondrocyte differentiation, as shown in Fig 5J). ADAMTS7 is a metalloproteinase that inhibits chondrocyte proliferation and differentiation through the inactivation of progranulin [29]. SNAI2 is a transcription factor that represses the differentiation of human epidermal progenitor cells and adipocytes [30, 31]. TGFBR1 is the receptor of TGF-β and represses osteogenic differentiation in rat bone marrow-derived mesenchymal stem cells [32]. Furthermore, heat map, pseudotime, and violin plots of *Adamts7*, *Snai2*, and *Tgfbr1* showed higher expression levels in Group 3 than in Group 5 (Fig 5K–5M), suggesting that these three genes are characteristically expressed in the early differentiation stage of ASCs.

### Age-associated changes in pseudotime

**Fig 6:**
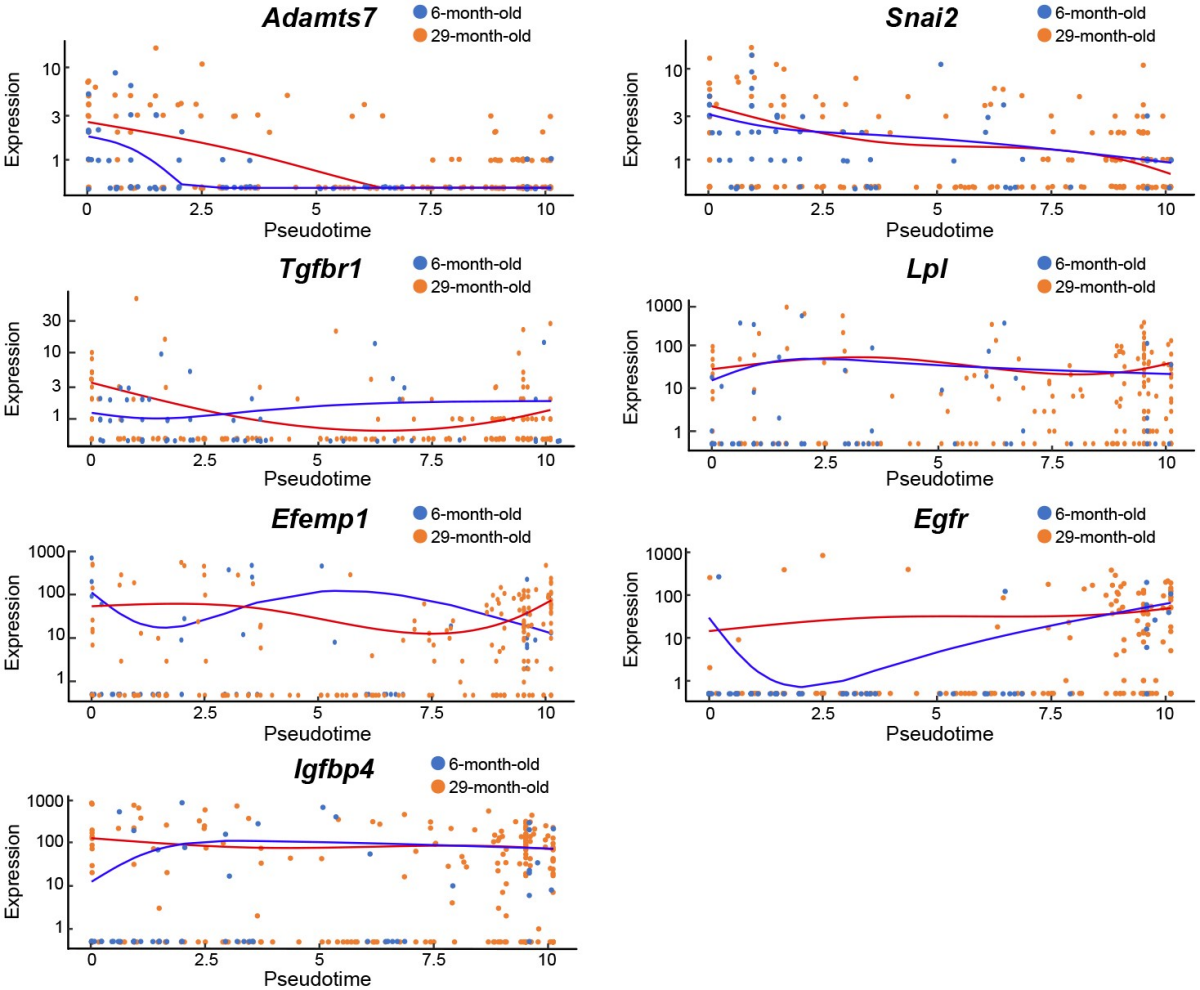
Age-associated changes in gene expression patterns. Kinetic plots showing the expression of *Adamts7*, *Snai2*, *Tgfbr1*, *Lpl*, *Efemp1*, *Egfr*, and *Igfbp4* over pseudotime are shown. The orange line indicates young cells (cell number, n = 61), and the blue line indicates old cells (cell number, n = 209).

We next assessed the age-associated changes in pseudotime for *Adamts7*, *Snai2*, and *Tgfbr1* and preadipocyte marker genes such as *Lpl*, *Efemp1*, *Egfr*, and *Igfbp4* between young and old mice. Age-associated changes with elapsed pseudotime were observed in the *Adamts7*, *Egfr*, and *Igfbp4* genes; however, there were no noticeable differences in the *Snai2*, *Tgfbr1*, *Lpl*, and *Efemp1* genes with aging (Fig 6).

### GSEA and PPI analysis

**Fig 7:**
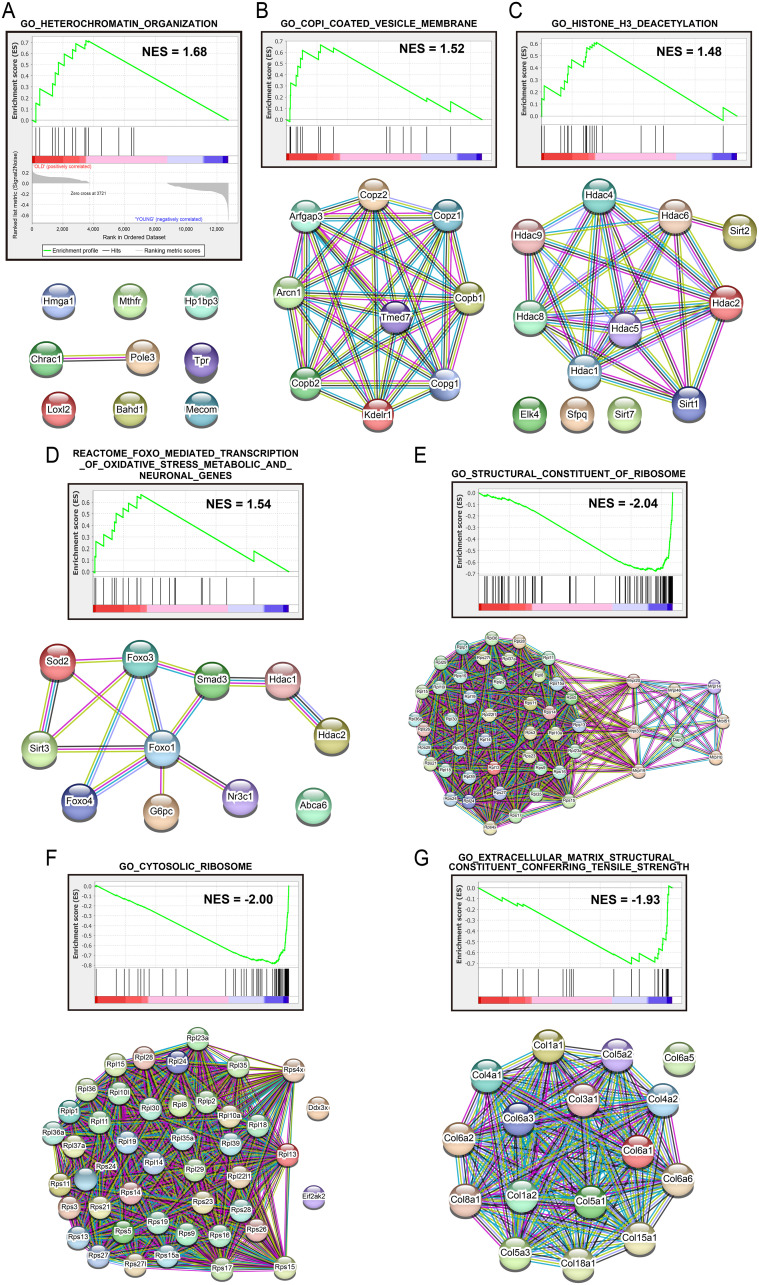
GSEA and PPI analysis between young and old ASCs. GSEA results showing the enrichment score (green line) and gene distributions (black line) among 12,729 genes rearranged according to the relative expression levels in Group 3 between young and old mice. Bars colored according to the gene distribution between the young (blue) and old (red) groups are shown. (A-C), (E-G) Results obtained using the GO public database (c5.all). (D) Results obtained using a curated public database (c2.all). All figures’ nominal p-values are lower than 0.05. (A–G) PPI among core enrichment genes of each gene set were analyzed using STRING. The confidence score cutoff for showing edges was set to ‘high’ (confidence score > 7.0). Edge colors indicated the type of evidence such as (i) co-expression (black), (ii) text-mining (yellow), (iii) biochemical/genetic data (“experiments”) (magenta), and (iv) previously curated pathway and protein-complex knowledge (“databases”) (cyan) of STRING.

Finally, we focused on Group 3 of the SVF because we considered Group 3 to represent the earliest differentiation stage of ASCs. We performed GSEA [22, 23] to compare young and old mice and found that four gene sets, corresponding to heterochromatin organization, coat protein complex I (COPI)-coated vesicle membrane, histone H3 deacetylation, and the forkhead box O (FOXO)-mediated transcription of oxidative stress metabolic and neuronal genes were enriched in old mice compared with young mice, and the corresponding normalized enrichment score (NES) was 1.68, 1.52, 1.48, and 1.54, respectively (Fig 7A–7D). These gene sets are listed in S5 Table. On the other hand, three gene sets, corresponding to the structural constituents of ribosomes, cytosolic ribosomes, and extracellular matrix structural constituents conferring tensile strength, were enriched in young mice compared with old mice, and the NESs of these sets were -2.04, -2.00, and -1.93, respectively (Fig 7E–7G). These gene sets are listed in S6 Table. Furthermore, we performed PPI analysis using STRING [25], and observed protein-protein interactions among the core enriched genes (Fig 7A–7G).

## Discussion

In this study, we performed a comprehensive single-cell transcriptome analysis to identify ASCs in SVFs from young and old mice and investigated the age-associated changes in ASC-related gene expression using various bioinformatic approaches. We distinguished three influential cell populations, classified as Groups 1, 3, and 5 out of eleven cell populations in the SVF, as putative ASC candidates, and we identified age-associated high gene expression levels of *Adamts7*, *Egfr*, and *Igfbp4* in the earliest differentiation stage of ASCs by pseudotime analysis. Moreover, four gene sets identified by GSEA, corresponding to heterochromatin organization, the COPI-coated vesicle membrane, histone H3 deacetylation, and the FOXO-mediated transcription of oxidative stress metabolic and neuronal genes, were more enriched in the early stage of ASCs from old mice than in those from young mice.

Three cell populations (Groups 1, 3, and 5) expressed highly typical ASC marker genes, *such as Gsn*, *Cxcl1*, *Col1a2*, *Col6a1*, *Mmp2*, and *Mmp14*, compared with the other eight groups, strongly suggesting that these three cell populations in the SVF were the most likely ASC candidates. In addition, the percentages of cells that were positive for the expression of these ASC marker genes were different among these three groups, indicating that these three groups represented different cell populations showing different gene expression patterns. Furthermore, although the percentages of cells that were positive for the expression of *Cxcl1* and *Mmp2* in Group 1 and *Gsn* in Group 3 in old mice were higher than those in young mice, no marked age-associated differences were observed for other genes or other groups. These results suggested that the change in the percentage of cells that were positive for the expression of ASC marker genes with aging was not particularly large.

Among a cohort of stem cell surface markers, stem cell antigen-1 (Sca1; Ly6A/E) was originally identified as a cell surface protein that allows the identification of hematopoietic stem cells [33, 34] and mesenchymal stem cells, including ASCs [35, 36]. Sca1/Ly6A is encoded by the *Ly6a* gene, the prototypical member of the Ly-6 superfamily, and the *Ly6a* gene has two alleles, *Ly6A* and *Ly6E* [34, 37]. Cross-reactive antibodies have been widely used to evaluate the expression of these proteins in stem cells, and these allelic variants are often referred to together as Ly-6A/E [38]. In this study, the clustering analysis of single-cell gene expression datasets from two different age groups did not reveal *Ly6a* and *Ly6e* genes as highly expressed genes in ASCs. Therefore, we originally checked *Ly6a* and *Ly6e* gene expression levels in Groups 1, 3, and 5 for comparison with the other eight groups distinguished from SVFs and found that *Ly6a* showed high expression levels in Groups 1, 3, and 5 (as indicated in A in S3 Fig); however, *Ly6e* showed higher expression levels in the other eight groups than in Groups 1, 3, and 5 (as indicated in B in S3 Fig). Thus, the regulation of *Ly6a* and *Ly6e* gene expression must differ in each cell type.

According to the preadipocyte and white adipocyte marker gene expression levels and t-SNE plots of Groups 1, 3, and 5, we estimated that Group 3 represented the earliest differentiation stage, exhibiting strong stem cell properties of ASCs, followed by Group 1, while Group 5 represented the most advanced differentiation stage. After building and drawing pseudotime paths using Monocle 3 [16], we identified three novel genes, *Adamts7*, *Snai2*, and *Tgfbr1*, which were expressed in Groups 3 and 5. These three genes are negative regulators of cell differentiation and were highly expressed in Group 3 compared with Group 5. ADAMTS7 *(Adamts7)* is a metalloproteinase that belongs to the ADAMTS family and inhibits chondrocyte proliferation and differentiation by inactivating progranulin, which a secreted protein in cultured ASCs [29, 39, 40]. However, it is still unclear how ADAMTS7 is involved in the differentiation and inactivation of progranulin in ASCs. In this study, high expression levels of *Adamts7* were observed in the early differentiation stage of ASCs, after which its expression levels decreased greatly in the advanced differentiation stage of ASCs according to the kinetic plot of the pseudotime analysis. These results strongly suggest that ADAMTS7 might function as an ASC differentiation suppressor to maintain the stemness of ASCs via the inactivation of progranulin. Interestingly, the high expression levels of *Adamts7* observed in the early differentiation stage of ASCs from old mice were not greatly reduced in the kinetic plot of the pseudotime analysis, suggesting that ASCs from old mice might show difficulty in progressing smoothly to cell differentiation when needed compared with young mice. SNAI2 (*Snai2*) is a transcription factor that represses the differentiation of human epidermal progenitor cells and adipocytes [30, 31]. Additionally, TGFBR1 (*Tgfbr1*) is the receptor of TGF-β and represses osteogenic differentiation in rat bone marrow-derived mesenchymal stem cells [32], but the relationship between TGFBR1 and ASC differentiation remains unclear. Concerning *Snai2* and *Tgfbr1*, we could not identify large age-associated changes in gene expression in the kinetic plot of the pseudotime analysis.

In this study, we identified age-associated changes in the pseudotime path for *Adamts7*, as described, and for *Egfr* and *Igfbp4* between young and old mice. EGFR (*Egfr*) is the receptor for epidermal growth factor (EGF) and activates several intracellular signal transduction pathways involved in cell differentiation and proliferation [41]. In cultured human ASCs, EGF treatment activates genes related to cell cycle progression, including the cell-cycle regulator cyclin D1 (*CCND1*), cyclin-dependent kinase 2 (*CDK2*), cyclin-dependent kinase 6 (*CDK6*), E2F transcription factor 1 (*E2F1*), and high mobility group AT-hook 1 (*HMGA1*), and adipogenesis-related genes such as *PPARγ*, peroxisome proliferator-activated receptor-γ coactivator-1α (*PGC1α*), and *C/EBPα* [42]. In this study, a high *Egfr* expression level was observed in the early differentiation stage of ASCs from both young and old mice; however, the *Egfr* expression level was greatly reduced only in the ASCs from young mice and then rapidly increased in the advanced differentiation stage of ASCs according to the kinetic plot of the pseudotime analysis. On the other hand, ASCs from old mice showed little change in *Egfr* expression levels according to pseudotime analysis. These results suggest that the ability to switch between the proliferation and differentiation of ASCs may have declined with aging. Additionally, IGFBP4 (*Igfbp4*) is highly expressed in adipocytes and osteoblasts and acts as an inhibitory binding protein of insulin-like growth factor 1 (IGF1), which is an important regulator of adipose tissue development *in vitro* [43–45]. In this study, the gene expression of *Igfbp4* was higher in ASCs from old mice than in those from young mice during the early differentiation stage. These results suggest that the expression of genes that control differentiation may be loosely regulated in ASCs from old mice. These pseudotime analyses revealed the expression patterns of several genes during the differentiation of ASCs into preadipocytes *in vivo* for the first time and clarified age-associated changes.

Moreover, we performed GSEA using only the transcriptome data of Group 3 because we considered this group to represent the earliest differentiation stage of ASCs from young and old mice and found that four gene sets, corresponding to heterochromatin organization, the COPI-coated vesicle membrane, histone H3 deacetylation, and FOXO-mediated transcription of oxidative stress metabolic and neuronal genes were enriched in the early differentiation stage of ASCs from old mice compared with those from young mice. Regarding the heterochromatin organization set, we found high mobility group AT-hook 1 (HMGA1), DNA polymerase epsilon 3, accessory subunit (POLE3), and chromatin accessibility complex subunit 1 (CHRAC1) in this gene set, as shown in S5 Table and Fig 7A. The CHRAC1/POLE3 heterodimer enhances ATP-dependent nucleosome sliding for chromatin remodeling and is involved in DNA double-strand break repair [46, 47]. HMGA1 is crucial for senescence-associated heterochromatic focus formation [48]. In the gene set related to the COPI-coated vesicle membrane, COPI is a master regulator of Golgi cisternal maturation and dynamics and consists of 7 subunits (α, β, β’, γ, δ, ε, and ζ) [49]. In this study, the α, β, γ, ε, and ζ subunits of COPI were identified in ASCs from old mice. These results suggest that aging causes structural destabilization and dysfunction of retrograde transport by COPI. Regarding histone H3 deacetylation, we found sirtuin 1 (SIRT1), sirtuin 2 (SIRT2), and histone deacetylase 6 (HDAC6) in the corresponding gene set. SIRT1 and SIRT2 are protein deacetylases targeting p53, DNA methyltransferase 1, and the FOXO family [50]. SIRT1 knockdown decreases human ASC proliferation and differentiation [51], and SIRT2 knockdown promotes 3T3-L1 preadipocyte differentiation into adipocytes [52]. PPI analysis revealed that HDAC6 interacts with SIRT1 and SIRT2, as shown in Fig 7C. HDAC6/SIRT1 attenuated nucleotide excision repair through deacetylation of replication protein A1 in eukaryotic cells [53]. Enriched CHRAC1, POLE3, SIRT1, and HDAC6 might suggest that DNA double-strand break repair processes are promoted in the early differentiation stage of ASCs from old mice. The FOXO-mediated transcription of oxidative stress metabolic and neuronal gene sets included FOXO1, FOXO3, FOXO4, FOXO6, and superoxide dismutase 2 (SOD2). PPI analysis revealed two interactions between FOXO1 and SOD2 and among FOXO1, FOXO3, and FOXO4, as shown in Fig 7D. FOXO1 and SOD2 regulate adipocyte differentiation [54, 55], whereas FOXO4 is involved in cell senescence through the inhibition of p53-induced apoptosis via direct binding and is a target for the development of senolytic drugs [56, 57].

Furthermore, three gene sets, corresponding to the structural constituents of ribosomes, cytosolic ribosomes, and extracellular matrix structural constituents conferring tensile strength, were enriched in young mice compared with old mice. Here, we found that the collagen type VI alpha (COL6A) family in the gene set of extracellular matrix structural constituents conferred tensile strength. Collagen type VI is a primary component of the white adipose tissue extracellular matrix [58] and plays important roles in adipocyte development through the self-renewal or differentiation of ASCs [59]. Deficiency of COL6A in knockout mice induces adipocyte hypertrophy and fragility of the white adipose tissue extracellular matrix, although the total fat weight is lower than in wild-type mice [59].

PPI analysis indicated no interactions between collagen type VI alpha 5 chain (COL6A5) and other members of the COL6A family of proteins, as shown in Fig 7G. However, the role of COL6A5 in ASCs remains unclear. In this study, although no age-associated differences were observed in the percentage of cells positive for the expression of *Col6a1*, the expression of other genes of the COL6A family may be attenuated with aging.

## Conclusions

Collectively, these results strongly suggest that with aging, the stringency of the regulation of the expression of some genes related to the differentiation of ASCs may not be maintained. These findings will greatly contribute to the development of regenerative medicine.

## Supporting information

S1 Fig Isolation of the SVF. Epididymal adipose tissues were removed from 6-month-old or 29-month-old male mice and digested by incubation with 2 mg/mL type I collagenase. The cell pellet was washed and then used as the SVF for single-cell transcriptome analysis.
(TIF)

S2 Fig Nx1-seq. Cells were allowed to settle into the wells of a polydimethylsiloxane slide via gravity. The slides were covered with a dialysis membrane and then incubated with a cell lysis solution containing detergents. After lysis, poly(dT) barcoded beads with bound cellular mRNA were collected in a microtube, and cDNA was synthesized with reverse transcriptase. The single-cell RNA sequencing results were read with a NextSeq 500/550 High Output v2 Kit.
(TIF)

S3 Fig ASC marker gene expression in SVFs. The gene expression levels of *Ly6a* and *Ly6e* in Groups 1, 3, and 5 combined (as indicated in A) (cell number, n = 461) and eight other groups (as indicated in B) (cell number, n = 825) are shown. Values are presented as the means ± SEMs. The statistical analysis was performed using the two-tailed Welch’s *t*-test. *p<0.001.
(TIF)

S1 Table Gene ontology terms of eleven groups in SVFs. (XLSX)

S2 Table A list of gene ontology terms and differentially expressed genes in eleven groups. (XLSX)

S3 Table A list of genes whose expression levels changed along with the elapsed pseudotime tested by Moran’s I. (XLSX)

S4 Table A list of genes in Module 6. (XLSX)

S5 Table Gene sets enriched in old mice compared with young mice. (XLSX)

S6 Table Gene sets enriched in young mice compared with old mice. (XLSX)

## Abbreviations

ASCs: adipose-derived stem cells
COPI: coat protein complex I
DAVID: Database for Annotation, Visualization, and Integrated Discovery
EGF: epidermal growth factor
FOXO: forkhead box O
GO: Gene Ontology
GSEA: gene set enrichment analysis
IGF1: insulin-like growth factor 1
MGI: Mouse Genome Informatics
NES: normalized enrichment score
Nx1-seq: next-generation 1-cell sequencing
PPI: protein–protein interaction
SEM: standard error of the mean
SVF: stromal vascular fraction
t-SNE: t-distributed stochastic neighbor embedding
UMAP: Uniform Manifold Approximation and Projection
