# Supplementary figures and images for: Age-associated changes in the transcriptomes of non-cultured adipose-derived stem cells from young and old mice assessed via single-cell transcriptome analysis
Source: PLoS One. 2020 Nov 25;15(11):e0242171. doi: 10.1371/journal.pone.0242171 (PMC7688117; doi:10.1371/journal.pone.0242171)

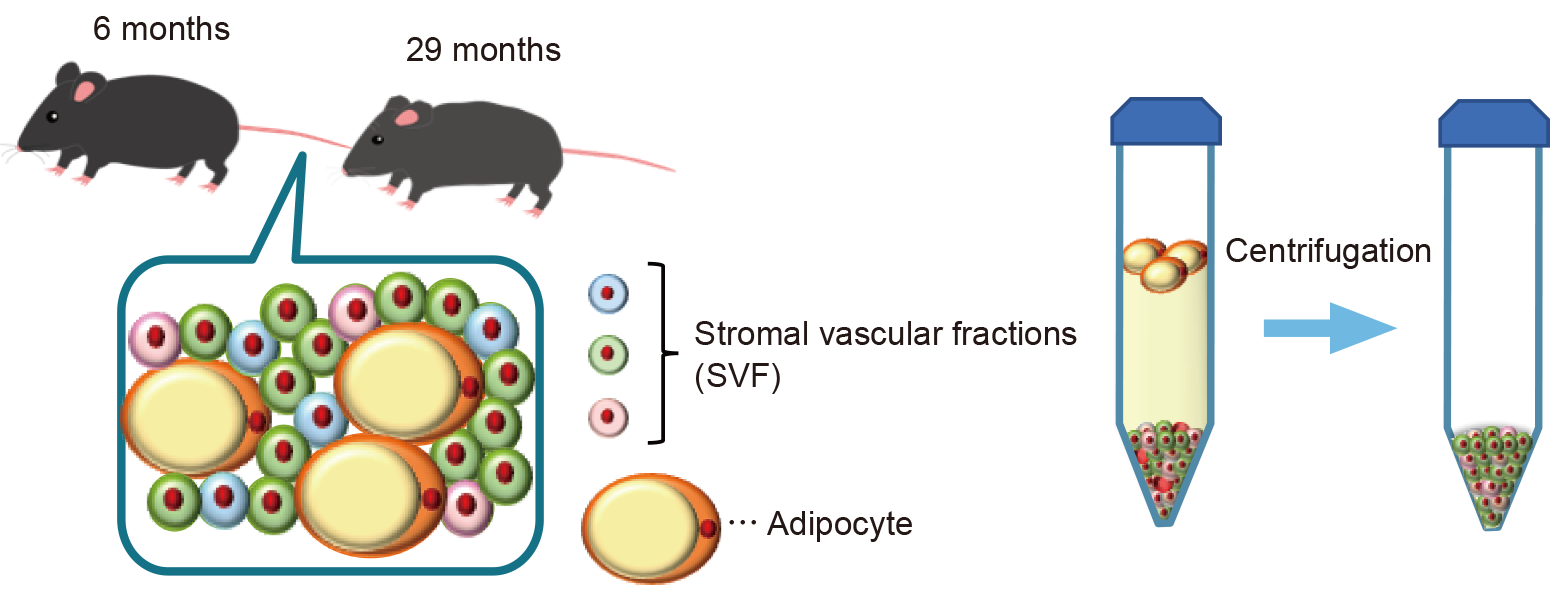

Supplement: S1 Fig — Epididymal adipose tissues were removed from 6-month-old or 29-month-old male mice and digested by incubation with 2 mg/mL type I collagenase. The cell pellet was washed and then used as the SVF for single-cell transcriptome analysis. (TIF) [file pone.0242171.s001.tif]

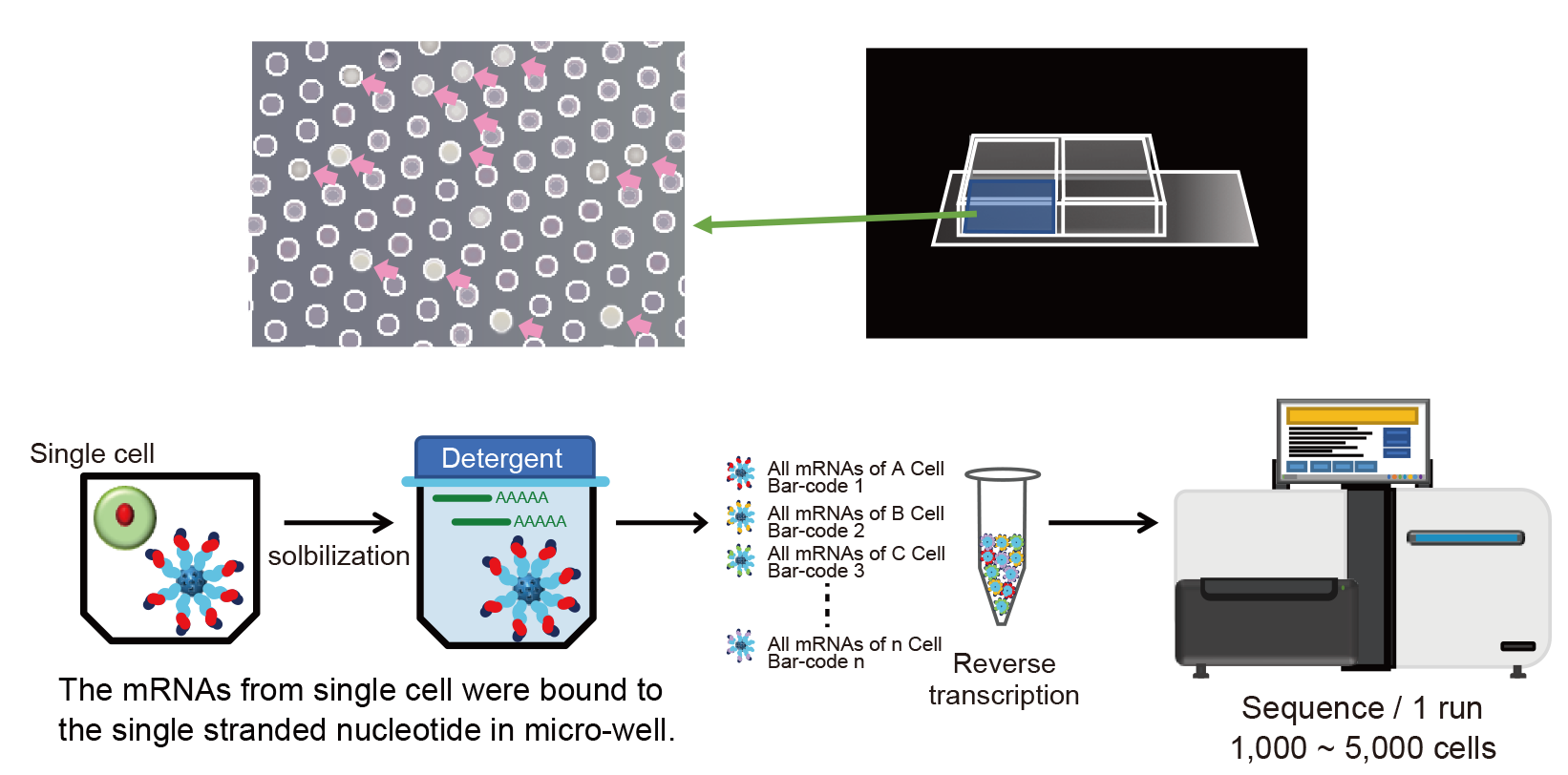

Supplement: S2 Fig — Cells were allowed to settle into the wells of a polydimethylsiloxane slide via gravity. The slides were covered with a dialysis membrane and then incubated with a cell lysis solution containing detergents. After lysis, poly(dT) barcoded beads with bound cellular mRNA were collected in a microtube, and cDNA was synthesized with reverse transcriptase. The single-cell RNA sequencing results were read with a NextSeq 500/550 High Output v2 Kit. (TIF) [file pone.0242171.s002.tif]

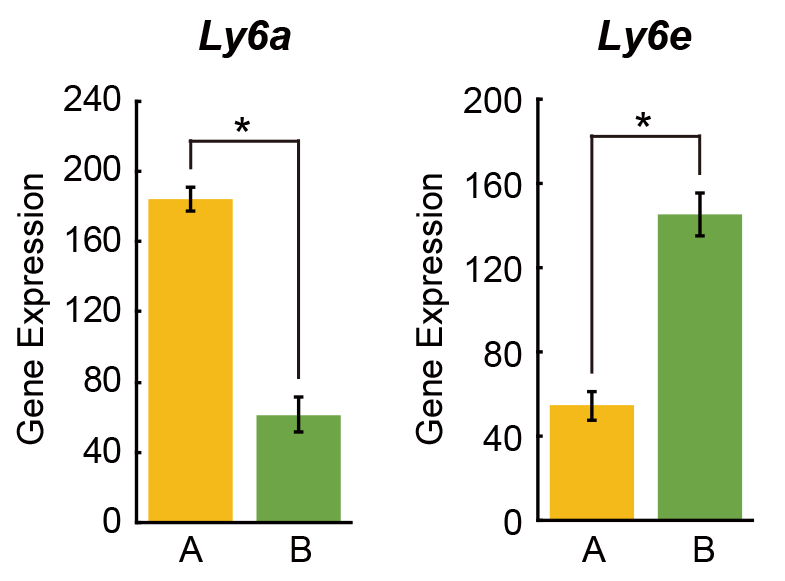

Supplement: S3 Fig — The gene expression levels of Ly6a and Ly6e in Groups 1, 3, and 5 combined (as indicated in A) (cell number, n = 461) and eight other groups (as indicated in B) (cell number, n = 825) are shown. Values are presented as the means ± SEMs. The statistical analysis was performed using the two-tailed Welch’s t-test. *p<0.001. (TIF) [file pone.0242171.s003.tif]
